# Surge Capacity and Capability. A Review of the History and Where the Science is Today Regarding Surge Capacity during a Mass Casualty Disaster

**DOI:** 10.3389/fpubh.2014.00029

**Published:** 2014-04-21

**Authors:** Randy D. Kearns, Bruce A. Cairns, Charles B. Cairns

**Affiliations:** ^1^Department of Surgery, University of North Carolina, Chapel Hill, NC, USA; ^2^Department of Emergency Medicine, University of North Carolina, Chapel Hill, NC, USA

**Keywords:** surge capacity, mass casualty incidents, disaster planning, disaster medicine, emergency medicine, emergency service, hospital, emergency medical services

## Abstract

Disasters which include countless killed and many more injured, have occurred throughout recorded history. Many of the same reports of disaster also include numerous accounts of individuals attempting to rescue those in great peril and render aid to the injured and infirmed. The purpose of this paper is to briefly discuss the transition through several periods of time with managing a surge of many patients. This review will focus on the triggering event, injury and illness, location where the care is provided and specifically discuss where the science is today.

## 100 Years Ago

The use of ambulances, the value of triage, and managing surge capacity at field hospitals were all at the forefront on an international stage in 1915–1918 (1). What was once described as; The Great World War, included weapons and arms seldom if ever used in combat such as submarines, airplanes, tanks, and chemical warfare (2). Managing the multitude of wounded soldiers included several more first time uses; motorized ambulance coaches in war and field hospitals with surgical care that went well beyond limb amputation (3).

As the war held worldwide attention, a novel influenza virus emerged and quickly spread reaching pandemic status. The virus silently moved through much of the world’s temperate climates killing millions of people including a disproportionately high number of youth (4). Civilian hospitals were quickly overwhelmed by the surge of infirmed patients. To meet the need, tent hospitals were erected and public buildings were adapted to be used as temporary field hospitals (5). The surge of patients overwhelmed the healthcare community. Unlike much of the previous experiences with war and disaster, the pandemic virus struck the clinicians as well. As clinicians fell ill, the additional struggle included managing the surge of patients with a depleted and frightened workforce.

Hospitals existed for centuries but discovery and invention during the late nineteenth and early twentieth century created a boom for improved healthcare and the facilities where that care was provided. The discoveries and invention included such things as antibiotics, radiography, surgical anesthesia, and adapting electronic inventions to monitor certain body functions. All contributed to the evolution and an ever growing complexity of health care.

War and other armed conflicts tested or drove new innovations in ambulance evacuation, triage, and field hospitals as witnessed in World War II. The Korean War included the first widespread use of helicopters to evacuate the wounded to field hospitals and set a new standard that would be replicated and improved upon in the conflicts and wars that followed. Triage standards and surgical field hospitals were well beyond anything previously seen near the battlefield.

The Cold War fueled funding and expansion of the civil defense programs, which included a medical surge component based on the threat of nuclear war (6). As the Vietnam War raged, a glaring gap in trauma and ambulance care was being debated in the United States leading to improved trauma systems, an organized Emergency Medical Service (EMS) system (7). Many of the civilian improvements were based on lessons learned in Vietnam. During the 1970s, these efforts to improve emergency care extended across much of the continents from Europe to Australia and North America.

However, as the threats faded, the wars ended, or the pandemic subsided, the interest for surge capacity also faded as well. As the twenty-first century approached, managing medical disasters in the civilian setting primarily focused on the occasional natural disaster or highway collision with dozens injured.

## Twenty-First Century

In the aftermath of the 11 September 2001 (9/11) attacks on the United States, American disaster planners rigorously reexamining the various aspects of medical disaster preparedness. Combining the 9/11 experiences with lessons learned from the international community and military planners who had dealt with and published their experiences involving a surge of patients during a disaster (8), a more coherent process began to emerge.

The 2004 manuscript by Hick et al. was the first of several that began to cohesively discuss the various and unique aspects of surge capacity and capability (9). While surge had occurred and was managed throughout the past century in the healthcare profession, what had changed was the structure, framework, and focus. Historically, surge management was based on instinctual behavior rather than institutional planning. Typically, the clinician who stepped into the command role for this sudden disaster was the emergency department physician, a staff physician such as a surgeon, or the emergency department charge nurse.

Catastrophic hurricanes in 2004 and 2005 left clinicians in vicarious situations as mechanical life support systems failed when the hospitals were damaged, or isolated by flooding and power loss. The emergence of a novel H5N1 influenza virus that was particularly deadly, without a proven vaccination compounded the anxiety. These real world events served as the impetus for a 2007 series of meetings focused on how to leverage staff, equipment, and treatment areas to assure emergency mass critical care (EMCC) was available for patients in a medical surge (10).

By 2009, several of these same researchers collaborated to produce what emerged as a key component for disaster planners; stratifying surge capacity and associating the escalating conditions with standard of care (11). This work emerged about the same time as the 2009 H1N1 influenza pandemic was spreading around the world. While not as widespread or deadly as the 1918 influenza pandemic, the potential ethical decisions, which loomed were significant. Although there was sufficient space and personnel to manage the surge of patients, the potential shortfall focused on how many ventilators were available to include the circuits and personnel needed to keep them going. How did that inventory match the potential influx of patients and if the patient numbers dramatically exceeded the capacity, what process was in place to decide who received the benefit of the ventilators (10, 12)?

The pandemic of 2009 highlighted a gap between the equipment available versus that which could be needed as well as adequate policies and processes being in place to aid in this decision-making process. While efforts were made to offer guidance for decision-makers in these grim ethical dilemmas, fortunately those difficult decisions were not required (12).

Additional guidelines were published (2012) from Hick et al. (13) to further aid the clinicians with how to allocate scarce resources relying on a “planned structured approach to include reactive and proactive triage guidelines” during a crisis surge capacity. The publication specifically identified six supply utilization strategies. They included; “prepare, conserve, substitute, adapt, reuse, and reallocate.” Furthermore, the triage focus for Hick et al. included a more specific focus on objective assessments and takes the steps necessary to avoid what is a natural tendency to over-triage patients during a disaster.

Confusing capacity and capability was also a point of focus as the science evolved. Having the capacity to manage patients in terms of space and supplies was insufficient if there was a lack of staff with the clinical capability to manage the patients. Or the space was adequate in size but lacked other important environmental requirements. Regardless, the convergent point included both capacity and capability. This paper will continue to describe surge in the context of capacity that is done only for the sake of simplicity.

## Surge Capacity in 2014; Staff, Space, and Supplies and the Standard of Care

As the research evolved, a clearer picture emerged to both understand and manage a surge of patients in the context of standards of care balancing the three aspects of *staff*, *space*, and *supplies* (pharmaceuticals, equipment, medical supplies). Each of these manuscripts build on the 2009 paper that stratified surge capacity into three defined categories: *Conventional, Contingency*, and *Crisis Surge Capacities* (11).

Key questions include; how does the disaster impact the *staff*, and is there sufficient staff on hand? Where is the care being provided in the facility (*space*)? Are there sufficient supplies, pharmaceuticals, and equipment (SPE) to manage the surge of patients (14)?

The three defined categories of Conventional, Contingency, and Crisis Surge Capacities are directly related to the usual and customary, standards of care a given patient should reasonably expect to receive any given day upon arrival at an emergency department or provided by an EMS agency. When there are more, or many more patients, plans and procedures should be in place to manage the surge of patients based on the staff, space, and supplies metrics previously discussed.

Using *conventional surge capacity* to describe a given event may be identified as “a busy day” with everyone doing what they typically do, with only limited supplement of additional staff, space, or supplies. Conventional surge may include holding staff over at the end of shift, bringing in extra ventilators from other floors in the hospital, and holding patients in a bed in the hall or other treatment rooms near the emergency department. Nevertheless, the traditional standard of care is intact for all patients.

Characteristics of *contingency surge capacity* include relying on space that is not typically used for emergent patients such as hospital conference rooms. Staffing will include clinicians with traditional credentials but may not be accustomed to managing acutely ill or critically injured patients. Examples may include physicians who are dermatologists, ophthalmologists, psychiatrists, pathologists, and may rely on nursing staff who now work in administration, or serve outside of the traditional clinical setting. Staff may include leveraging just-in-time (JIT) training to create a force multiplier [Israel demonstrated a 10:1 ratio of trained burn nurse to other nurses using a JIT approach (15)]. Supplies are limited and in some cases substitute medications or fluids are used due to insufficient supply for every patient’s needs. The most unpredictable limitation is the availability of and access to supplies, equipment, and pharmaceuticals needed (16).

*Crisis surge capacity* occurs with an event that overwhelms the hospital with care being provided in spaces that may be outside of the structure of the hospital such as tents erected in the parking lot, or adjacent medical office buildings, fitness centers, etc. Staffing and supplies are based on whatever is available with staffing including any and all willing and who can help during the disaster. The supplies may be sources from alternate locations such as the local drug store, disaster equipment caches, or off label use of items. Triage may include deciding who can be placed on a ventilator that is in short supply. Certainly, this is the direst of circumstances and every effort should be made to minimize the time the event is operating under these circumstances (17).

Crisis Surge Capacity implies the practice of care outside the traditional standard of care and should be avoided or alleviated as soon as reasonably possible. During the planning process, it is an excellent time to involve whoever is responsible for ethical policy review to assure all involved understand the latitude that may be needed to manage the disaster.

## Alternative Staff Resources, Just-in-Time Training, and Force Multiplier

One strategy used to expand the staffing resources during a crisis surge includes relying on disaster medical responders from other communities or non-traditional personnel to aid in managing the surge of patients. The US approach includes state and federalized disaster teams within a state system or the National Disaster Medical System (NDMS) (18). Depending on the size and scope of the disaster as well as local and in country resources, international response has made a significant impact with disasters such as the aftermath of the 2010 Haitian Earthquake that left hundreds of thousands injured as well as hundreds of thousands dead (19, 20).

Other strategies include relying on force multipliers through JIT training utilizing personnel who have the aptitude to quickly learn, adapt, and assist. Cross training prior to the disaster can boost personnel pools in preparation for a disaster but may not be a viable option for some organizations. Military strategies offer excellent examples of leveraging manpower not routinely considered clinicians, with JIT training and grouped with medically trained team leaders to manage larger numbers of patients (21, 22). For specialized advice, telemedicine may also be used to augment staff and provide expert assistance “virtually” so long as technology can support the effort (23).

## Recent Developments and Getting to Surge Equilibrium (Balance)

By 2012, the research was focused on either the scene of a disaster or the location where patients were being transported/treated. Another publication by Hick et al. (13) included a more specific emphasis including special considerations such as “specific events outside of the usual clinical resources.” Examples for what is described as special considerations include: pediatrics, burn injured patients, or patients needing decontamination. The response to the surge with staff, space, and supplies was intended to rely on whatever was available to meet the patient needs until the ongoing needs and those of newly arriving patients was being met by resources involved in the response. Additional research also discusses the value of transportation resources needed to partially alleviate stress caused by the surge of patients (24).

Creating this balance includes understanding what is available and what is needed in the context of the ongoing presentation of patients related to the disaster (13, 16). In certain theoretical circumstances where the probability is very low but the consequences are very high, such as in the aftermath of the detonation of an improvised nuclear device, patient needs will far outstrip resources in the immediate and contiguous areas (25, 26).

**Figure 1:**
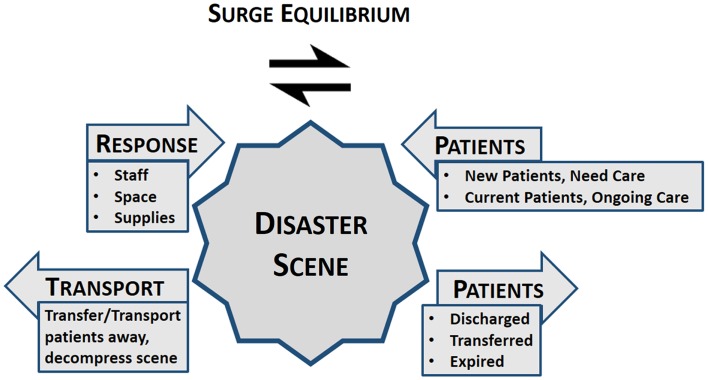
**Surge equilibrium: all competing influences of the disaster are balanced at the point of where the patients managed, disaster scene or at the hospital**.

As the disaster unfolds, there are indicators, which suggest when things are beginning to stabilize. In the midst of the disaster, it is difficult to know when this is taking place. Nevertheless, a recent addition to the academic literature offered an explanation of reaching the balance where sufficient staff, space, and supplies are now available at the disaster location to manage the ongoing patient needs. This balance [*Surge Equilibrium* (Figure 1)] is reached when the response of staff, space, and supplies to the point where the surge is being managed, as well as transports away, matches the patients’ needs. The patient volume side of the equation includes those newly arriving and those with ongoing needs, but is decreased when patients have been transferred, managed and discharged, or died. Thus, the equilibrium (balance) is reached when the numbers of newly arriving patients as well as those with ongoing needs are being met on a steady and predictable basis by the staff, space, and supplies including transfers away from the disaster scene.

## Conclusion

Managing a surge of patients during a disaster requires; planning, speed, repetition, training, and simplicity. Disaster plans should build upon daily and preplanned activities. If the use of triage tags is advocated and identified in a plan, but used only during a mass casualty incident, the lack of familiarity can lead to failure. If response spaces and supplies for the surge of patients are never identified and staff is not trained to manage the surge, success when this disaster strikes is unlikely.

Clinicians are accustomed to managing patients based on the traditional standard of care. However, can they recognize the signs that response resources are being overwhelmed by patients’ needs during an ever escalating event? When this happens, what is the plan and what are the processes to maintain control of the incident?

When disaster strikes, EMS is typically the first source of information and the first to start the process of managing the surge of patients. As the disaster unfolds, this will make transition to the emergency department clinicians who will see the first wave of patients to include those who self-evacuate as well as those who are transported by EMS.

Planning and preparedness activities will minimize the confusion and needless loss of life, and maximize the response to the disaster to include the allocation of resources. These activities should also identify potential failure points and whom to call for assistance before being overwhelmed. While creativity and luck may contribute to a successful outcome, for the best outcomes, the leaders during the disaster must be involved in the planning and preparedness activities before the disaster.

## Conflict of Interest Statement

The authors declare that the research was conducted in the absence of any commercial or financial relationships that could be construed as a potential conflict of interest.
